# Supplementary material for: Comprehensive Annotation and Functional Exploration of MicroRNAs in Lettuce
Source: Front Plant Sci. 2021 Dec 24;12:781836. doi: 10.3389/fpls.2021.781836 (PMC8739914; doi:10.3389/fpls.2021.781836)
Supplement: Supplementary file 2 [file Data_Sheet_8.ZIP › GSM2230748.plot/Lsa-miRN1652a_Lsat_1_v5_gn_7_48060.1_110_TPlot.pdf]

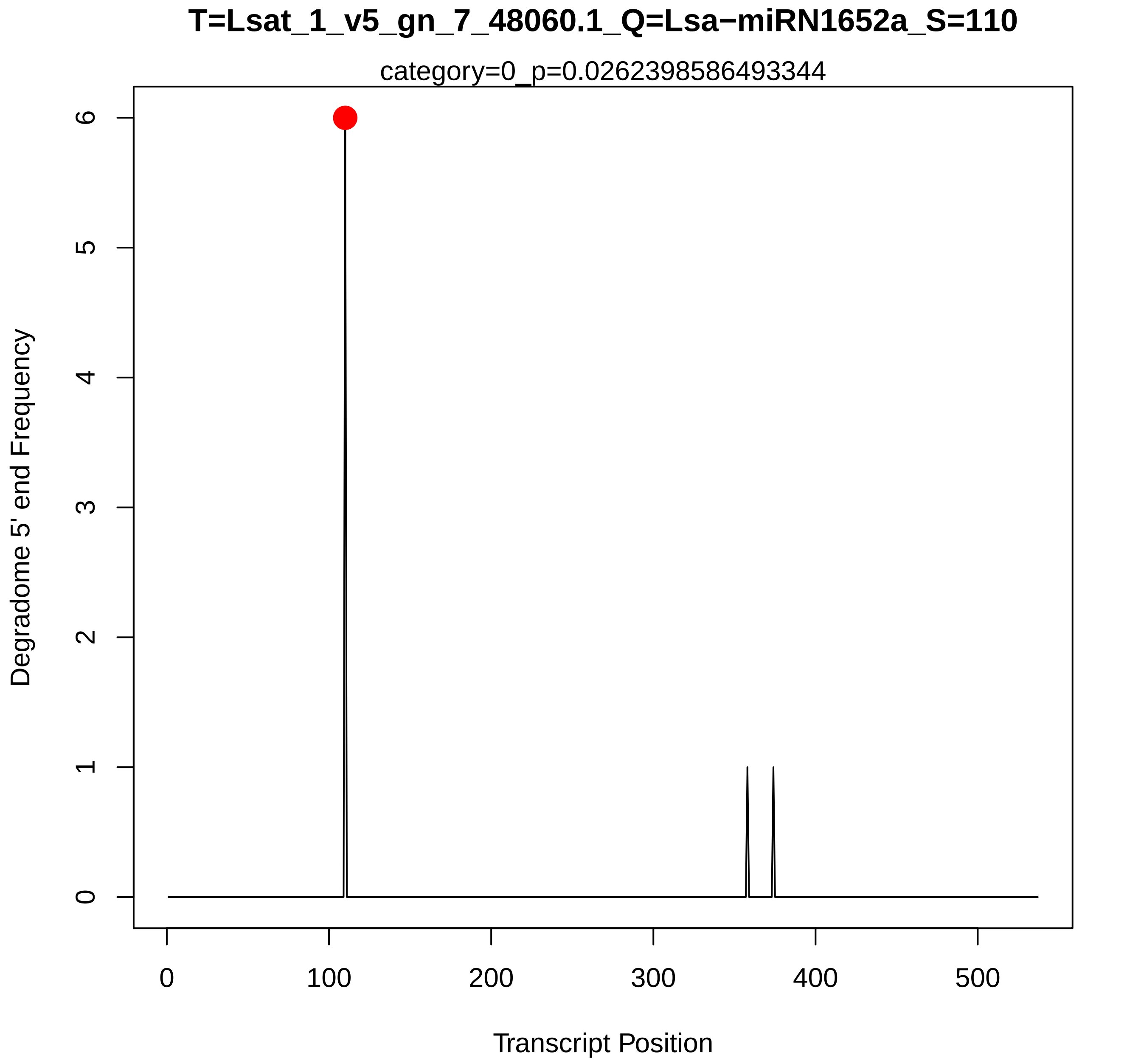

T=Lsat_1_v5_gn_7_48060.1_Q=Lsa−miRN1652a_S=110
category=0_p=0.0262398586493344
Degradome 5' end Frequency
0
1
2
3
4
5
6
0
100
200
300
400
500
Transcript Position
